# Impact of smart healthcare on the physical and mental health of older adults Chinese patients with chronic diseases under the background of “Internet Plus”: analysis of data from the China family panel studies

**DOI:** 10.3389/fpubh.2025.1475456

**Published:** 2025-09-08

**Authors:** Fanbao Xie, Xinyi Liang, Xin Guan, Peng Ji, Xiaojing Cao, Jiarong Liao

**Affiliations:** ^1^School of History and Public Administration, Yancheng Teachers University, Yancheng, China; ^2^Institute of Yancheng Regional Culture and Social Governance, Yancheng, China; ^3^School of Public Administration, Guangzhou University, Guangzhou, China; ^4^Guangzhou Xinhua University, Dongguan, China; ^5^Researching Center of Social Security, Wuhan University, Wuhan, Hubei, China

**Keywords:** smart healthcare, older adults chronic disease, physical and mental health, internet, depression

## Abstract

**Objective:**

This study was to investigate the impact of smart healthcare on the physical and mental health of older adults chronic disease patients in China.

**Methods:**

Data from 6,092 chronic disease patients were collected from the 2022 China family panel studies (CFPS). Participants were rolled into two groups based on whether they utilized Internet Plus-based smart healthcare technology for chronic disease management: the usage group (*n* = 2093) and the non-usage group (*n* = 3,999). A comparison was conducted between the two groups in terms of activities of daily living (ADL) scores, self-rated health (SRH), and the center for epidemiologic studies depression scale (CES-D). Furthermore, a multivariate logistic regression analysis was employed to identify the risk factors influencing the physical and mental health of patients in the usage group.

**Results:**

Post-intervention, the SRH and ADL scores of the usage group were greatly superior to those of the non-usage group, while the CES-D scores were greatly lower (*p <* 0.05). Samples with chronic disease duration exceeding 10 years demonstrated the lowest SRH and ADL scores and the highest CES-D scores (*p <* 0.05). Similarly, samples experiencing chronic pain exhibited the lowest SRH and ADL scores and the highest CES-D scores (*p <* 0.05). Samples with an income level below 10,000 yuan per annum demonstrated the lowest SRH and ADL scores and the highest CES-D scores (*p <* 0.05). Additionally, individuals with an educational attainment of primary school or below exhibited the lowest SRH and ADL scores and the highest CES-D scores (*p <* 0.05). Chronic disease duration exceeding 10 years and chronic pain were identified as significant risk factors affecting ADL and CES-D scores. Chronic disease duration exceeding 10 years, chronic pain, an income level below 10,000 yuan per annum, and an educational attainment of primary school or below were identified as risk factors for SRH scores (*p <* 0.05).

**Conclusion:**

With the assistance of Internet Plus-based smart healthcare, the physical and mental health of older adults chronic disease patients in China can be effectively improved. However, the influence of longer chronic disease duration, chronic pain, low income, and low educational attainment on the effectiveness of smart healthcare assistance should be considered.

## 1 Introduction

With the intensifying trend of global population aging, the health issues of the older adults population are becoming increasingly prominent. According to data from the *National Bureau of Statistics of China*, by the end of 2020, the population aged 60 and above in China had surpassed 260 million, accounting for 18.7% of the total population (1). The high prevalence of chronic diseases among the older adults has emerged as a significant public health challenge. Chronic diseases such as diabetes, hypertension, and heart disease are characterized by prolonged courses and complex conditions, necessitating long-term treatment and management. These diseases impose a substantial burden on patients, their families, and social healthcare resources (2–4). Enhancing the quality of life for older adults patients with chronic diseases and alleviating the medical burden have thus become urgent issues that need to be addressed.

The core concept of Internet Plus lies in the deep integration of information and communication technology (ICT) with traditional industries, aiming to restructure production factors, innovate service models, and enhance societal efficiency. In the healthcare sector, “Internet Plus” is specifically manifested through the integration and application of technologies such as telemedicine, mobile health applications (apps), wearable devices, AI-assisted diagnostics, and big data analytics. These technologies significantly optimize the entire process of chronic disease management, including disease monitoring, personalized interventions, and patient education, through real-time data collection, intelligent analysis, and remote collaboration. Smart healthcare, as the application of Internet Plus in the medical field, has rapidly developed and become widely adopted, offering new possibilities for chronic disease management (5). Smart healthcare encompasses not only telemedicine, online health consultations, and electronic health record management, but also includes applications such as intelligent device monitoring and health data analysis. These technologies provide comprehensive support for the prevention, diagnosis, treatment, and rehabilitation of chronic diseases (6). Despite the promising prospects of smart healthcare in chronic disease management, its actual effectiveness in older adults patients with chronic diseases remains to be further explored. Existing research predominantly focuses on the development and application of smart healthcare technologies, with relatively few studies specifically examining their impact on the physical and mental health of older adults chronic disease patients (7). Most of the current literature emphasizes the role of smart healthcare in disease monitoring and healthcare resource allocation (8, 9), while research on its effects on improving the psychological well-being and quality of life of older adults patients is comparatively lacking.

This study was to investigate the impact of smart healthcare on the physical and mental health of older adults chronic disease patients in China, based on data from the China family panel studies (CFPS). The CFPS dataset encompasses extensive household and individual information, including health status, utilization of medical services, and socio-economic conditions (10), providing a robust data foundation for this research. Through in-depth analysis, this study aimed to fill the existing research gap and provide theoretical and empirical support for the development of smart healthcare and older adults health management.

## 2 Path analysis

**Figure 1:**
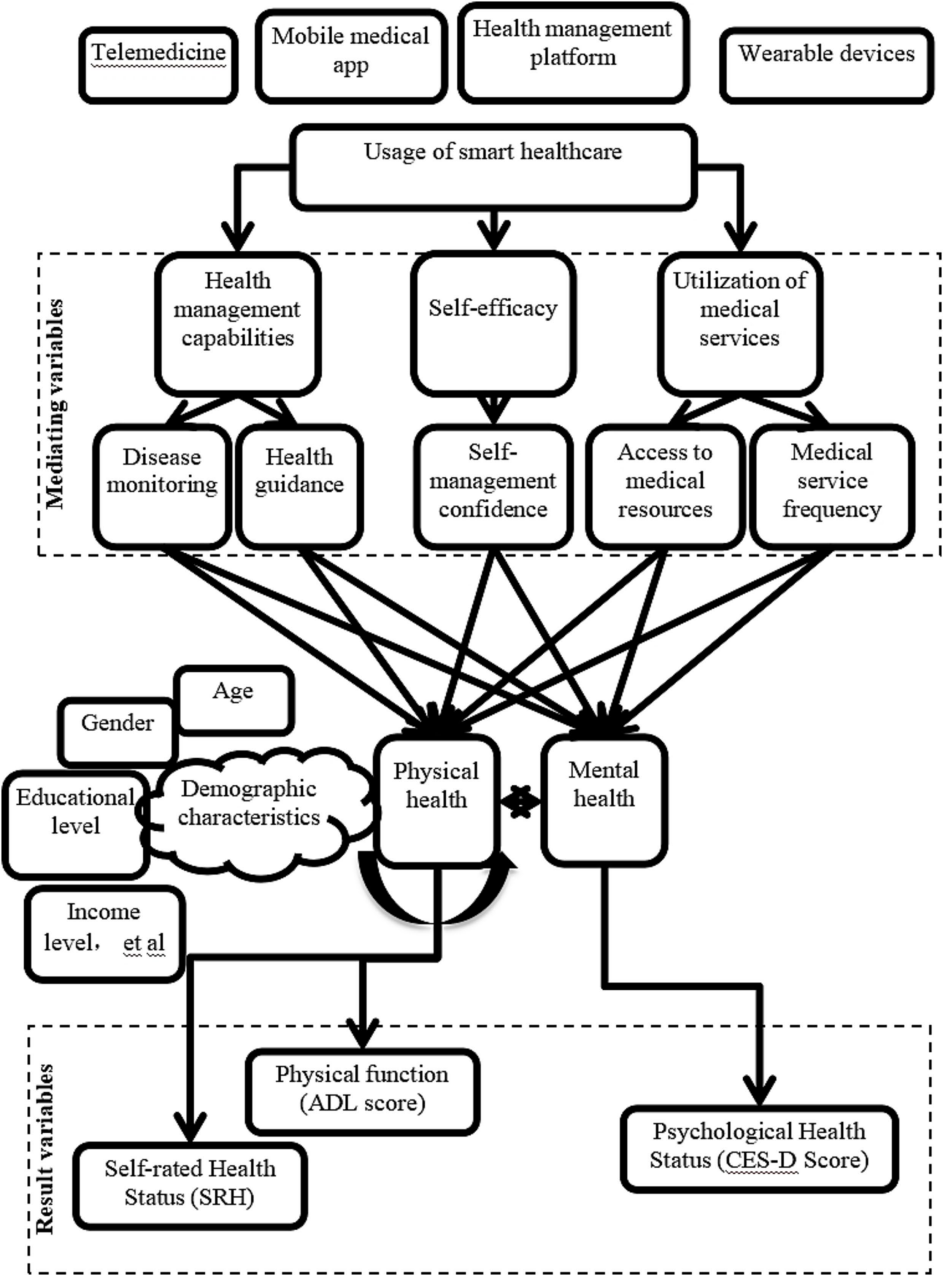
Pathway diagram of smart healthcare’s impact on the physical and mental health of older adults chronic disease patients.

To comprehensively understand the impact of smart healthcare on the physical and mental health of older adults chronic disease patients, a detailed pathway diagram was constructed (Figure 1). This diagram elucidates how smart healthcare influences the physical and psychological health of older adults chronic disease patients through various mechanisms.

“Internet Plus”-driven smart healthcare is as follows. Telemedicine, mobile healthcare apps, health management platforms, and wearable devices are smart healthcare tools that influence patients’ health management capabilities, self-efficacy, and utilization of medical services by providing real-time medical services and health monitoring.

Mediating variables include: (1) Health management capability: (a) Condition monitoring: continuous monitoring of patient health data through smart devices and platforms allows for timely detection and management of conditions; (b) Health guidance: personalized health advice and alert services help patients develop reasonable health plans. (2) Self-efficacy: (a) Self-management confidence: with the support of smart healthcare, patients exhibit increased confidence and proactivity in managing their health. (3) Utilization of medical services: (a) Access to medical resources: smart healthcare platforms enable patients to access medical resources more conveniently; (b) Frequency of medical services: the convenience of smart healthcare increases the frequency of patient visits, ensuring timely medical intervention.

Moderating variables include demographic characteristics (such as age, gender, education level, and income level), which moderate the effectiveness of smart healthcare utilization, with patients of different characteristics benefiting to varying degrees. The type of smart healthcare service also affects patients’ health outcomes differently, with specific impacts on physical and mental health varying across different types of smart healthcare services.

Outcome variables include: (1) Physical health status: through improving health management capability, self-efficacy, and increasing utilization of medical services, smart healthcare helps enhance patients’ physical functioning (activities of daily living (ADL) scores) and self-rated health (SRH) scores; (2) Psychological health status: smart healthcare reduces patients’ negative emotions (such as anxiety, depression), thus enhancing patients’ psychological well-being, as measured by the center for epidemiologic studies depression scale (CES-D).

## 3 Research methods

### 3.1 Data sources

This study utilized data from the 2022 CFPS. Organized by the *China Social Science Survey Center* of Peking University, the CFPS baseline sample covers 25 provinces nationwide, demonstrating high representativeness. The survey questionnaire underwent multiple rounds of validation and refinement to ensure its scientific rigor. Through rigorous reliability and validity testing, the questionnaire demonstrated a high Cronbach’s alpha coefficient of 0.96, indicating excellent item discrimination. Item and validity analyses revealed good fit indices, confirming the high authenticity and reliability of the data. Overall, these findings provide a solid foundation for the study.

### 3.2 Research object

The study extracted adult data from 25 provinces in the national dataset of CFPS 2022, totaling 6,092 samples for research analysis.

Inclusion criteria: (1) individuals aged 60 and above; (2) individuals diagnosed with chronic diseases such as diabetes, hypertension, and heart disease; (3) all underwent assessments of physical function (ADL score), self-rated health (SRH), and psychological health status (CES-D score).

Exclusion criteria: individual samples with missing data were excluded.

### 3.3 Research content

From the 6,092 survey responses, the usage of Internet Plus-based smart healthcare among patients was assessed. The samples were rolled into two groups: the usage group (those utilizing Internet Plus-based smart healthcare technology for chronic disease management) and the non-usage group (those not utilizing Internet Plus-based smart healthcare technology for chronic disease management). The criteria for smart healthcare technology usage were defined as follows: patients engaged in chronic disease management via telemedicine (≥1 session/month), mobile health apps (≥3 logins/week), health management platforms (≥1 data sync/week), or wearable devices (≥5 days/week wear). Usage frequency was cross-verified using platform backend data and questionnaire responses, and samples with user adherence below 80% were excluded.

### 3.4 Indicator collection

In conjunction with pathway analysis, the key indicators to be collected in this study included:

1. Physical and mental health indicators include self-rated health (SRH), psychological health score (CES-D), and physical functioning score (ADL). The primary data to be collected included the physical and mental health indicators of chronic disease patients in both the usage and non-usage groups, before and after a two-year intervention.
2. Smart healthcare utilization, including information regarding whether patients utilized telemedicine, mobile healthcare apps, and other smart healthcare services, was collected through questionnaire surveys, along with details regarding the specific types of smart healthcare services utilized.
3. Control variables, including basic demographic information such as gender, age, duration of chronic disease, types of chronic diseases, income, education level, and types of smart healthcare services utilized, were collected as control variables.

### 3.5 Statistical methods

The data in this study were processed and analyzed using *SPSS 20.0* and *Stata 17.0*. Descriptive statistical analysis was first performed to examine the sample characteristics, with categorical data presented as percentages and analyzed using the chi-square test. Continuous data were expressed as mean ± standard deviation and analyzed using independent t-tests. Subsequently, multivariate logistic regression analysis was conducted to identify the risk factors affecting the physical and mental health of patients in the intervention group. To address baseline differences between the control and intervention groups (e.g., ADL, SRH, and CES-D scores) and socioeconomic disparities (income, education level, chronic disease types), covariates were adjusted for in the regression analyses.

To control for potential endogeneity issues (such as the bidirectional causal relationship between the use of smart healthcare and health outcomes), this study further employs instrumental variable analysis (IVA) for evaluation. The “internet penetration rate in the province of the household (provincial-level statistical data for 2023)” and “whether the community is equipped with a smart healthcare service demonstration site (from the CFPS community database)” were selected as instrumental variables. Two-stage least squares (2SLS) regression was applied. The instrumental variables must satisfy the conditions of relevance (significant correlation with smart healthcare usage) and exogeneity (uncorrelated with the error term), which were validated using the weak instrument test (Cragg-Donald F-statistic >10) and the over-identification test (Hansen J test *p* > 0.05). Panel data for the years 2021–2023 from CFPS tracking data were used to construct the dataset, and a fixed-effects model was employed to control for individual heterogeneity in analyzing the dynamic impact of smart healthcare usage on health outcomes. Robustness checks were also conducted, including variable replacement (replacing self-reported health \[SRH] with objective health indicators, such as the incidence of chronic disease complications), subgroup regression (repeating the analysis by gender and urban–rural classification), and propensity score matching (PSM) using 1:1 nearest-neighbor matching to balance baseline characteristics between the treatment and control groups. All analyses considered a significance level of *p* < 0.05. To mitigate potential hidden bias, this study employed intention-to-treat (ITT) analysis and utilized two methods for handling missing data: multiple imputation (MI) and last observation carried forward (LOCF). The imputation models incorporated all core covariates and auxiliary indicators.

## 4 Results

### 4.1 Dropout and ITT analysis results

During the two-year intervention period, 312 participants (5.12% of total sample) were lost to follow-up, including 89 deaths (42 in user group, 47 in non-user group) and 223 dropouts (141 in user group, 82 in non-user group). The between-group attrition difference was significant (χ^2^ = 98.3, *p* < 0.001), with higher attrition in users (183/2093, 8.74%) than non-users (129/3999, 3.22%). Compared to completers, user-group dropouts had lower baseline ADL scores (60.2 ± 8.1 *vs.* 65.3 ± 10.3, *p* = 0.010) and higher CES-D scores (52.3 ± 9.8 *vs.* 48.1 ± 10.0, *p* = 0.030), but no significant SRH difference (2.87 ± 0.64 *vs.* 2.92 ± 0.61, *p* = 0.421). Non-user dropouts showed no baseline differences in ADL, CES-D or SRH versus completers (all *p* > 0.05). ITT analyses using MI and LOCF yielded consistent results with primary analysis: SRH: MI: *β* = 0.41 (95%CI: 0.07–0.75, *p* = 0.017); LOCF: *β* = 0.39 (95%CI: 0.06–0.72, *p* = 0.022); ADL: MI: *β* = 4.05 (95%CI: 0.86–7.24, *p* = 0.013); LOCF: *β* = 3.92 (95%CI: 0.69–7.15, *p* = 0.019); CES-D: MI: *β* = −2.81 (95%CI: −5.31 to −0.31, *p* = 0.028); LOCF: *β* = −2.75 (95%CI: −5.22 to −0.28, *p* = 0.031).

### 4.2 Statistics on basic information of two groups of patients

The study collected and analyzed data from 6,092 samples, including the number of chronic disease patients in both the usage and non-usage groups, as well as basic demographic information such as gender, age, duration of chronic disease, types of chronic diseases, income, education level, marital status, and types of smart healthcare services utilized. Specific details are presented in Table 1. Of these, the usage group comprised 2,093 samples, while the non-usage group comprised 3,999 samples, indicating a higher number of older adults chronic disease patients not utilizing Internet Plus-based smart healthcare assistance. Statistical analysis indicated neglectable differences in the distribution of gender, age, duration of chronic disease, types of chronic diseases, income, education level, and other demographic characteristics between the usage and non-usage groups (*p* > 0.05). To control for potential confounding effects of socioeconomic disparities, subsequent multivariate regression models and 2SLS analyses adjusted for covariates including income, education level, and chronic disease types to prevent overestimation of intervention effects due to baseline differences.

**Table 1:** Basic data statistics.

Variable	Non-usage group	Usage group	*p*
Sample size (*n*/%)	3,999	2093	–
Gender (*n*/%)			0.678
Male	2,000 (50%)	1,000 (47.8%)	
Female	1,999 (50%)	1,093 (52.2%)	
Age (years old)			0.845
Range	60–80	60–80	
Mean age	69.5 ± 15.2	71.3 ± 15.3	
Chronic disease course (years)			0.912
<1 year	500 (12.5%)	250 (12%)	
1–5 years	1,500 (37.5%)	780 (37.3%)	
6–10 years	1,200 (30%)	650 (31%)	
>10 years	799 (20%)	413 (19.7%)	
Types of chronic diseases (*n*/%)			0.733
Diabetes	1,000 (25%)	510 (24.4%)	
Cardiovascular disease	1,500 (37.5%)	800 (38.2%)	
Chronic pain	600 (15%)	300 (14.3%)	
Chronic respiratory system diseases	400 (10%)	220 (10.5%)	
Others	499 (12.5%)	263 (12.6%)	
Income (*n*/%)			0.854
Below 10,000 yuan	500 (12.5%)	250 (12%)	
10,000–50,000 yuan	1,500 (37.5%)	800 (38.2%)	
50,000–100,000 yuan	1,000 (25%)	520 (24.8%)	
Over 100,000 yuan	999 (25%)	523 (25%)	
Education level (*n*/%)			0.679
Primary school and below	500 (12.5%)	300 (14.3%)	
Junior high school	1,000 (25%)	520 (24.8%)	
High school/vocational school	1,200 (30%)	600 (28.7%)	
Junior college	799 (20%)	413 (19.7%)	
Undergraduate or above	500 (12.5%)	260 (12.4%)	
Types of smart healthcare services (*n*/%)			–
Telemedicine		523 (25%)	
Mobile medical app		780 (37.3%)	
Health management platform		373 (17.8%)	
Wearable devices		297 (14.2%)	
Others		120 (0.7%)	

### 4.3 Analysis of differences in results between the experimental and control groups

**Figure 2:**
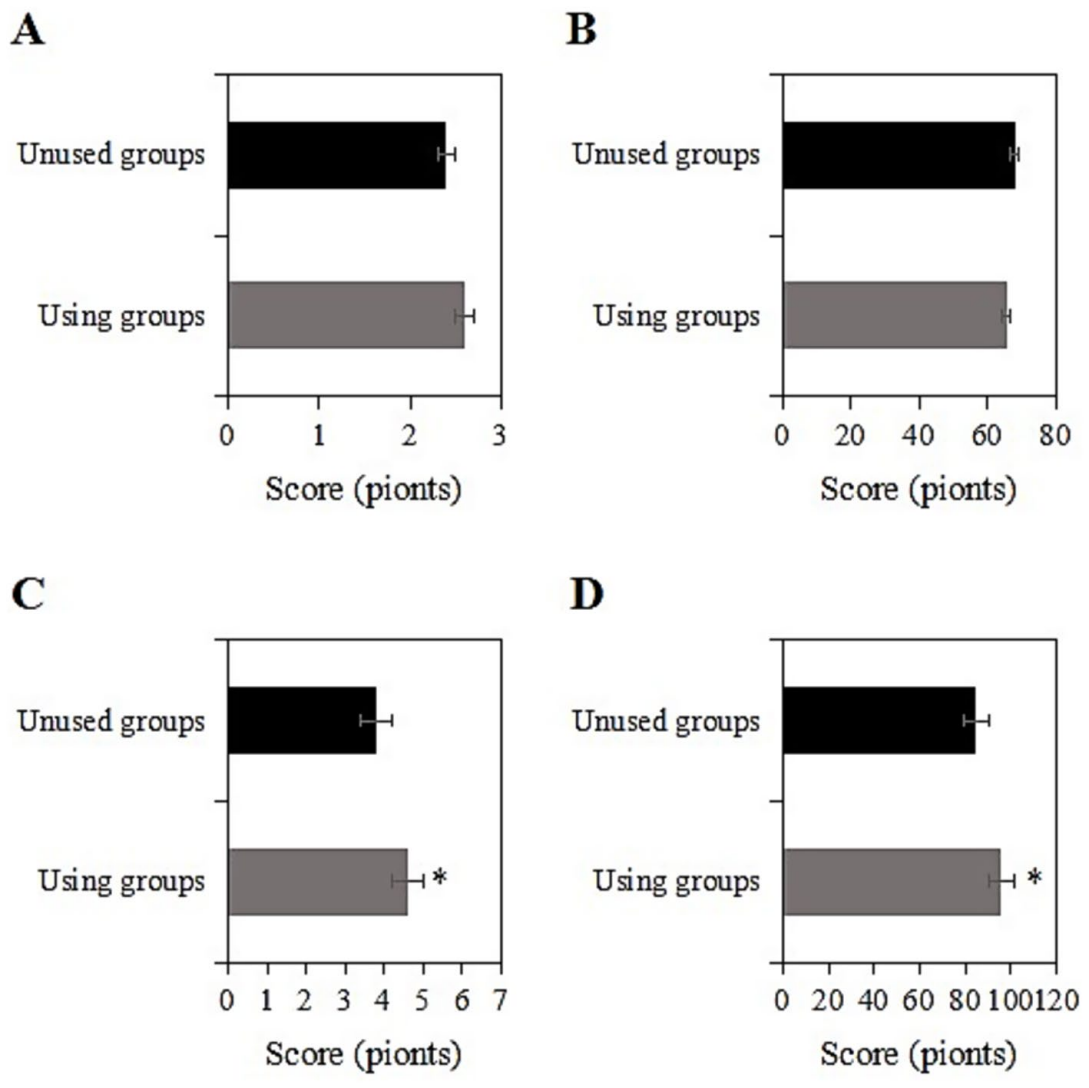
Comparison of SRH and ADL scores before and 2 years after intervention [**(A,B)** SRH and ADL scores before intervention; **(C,D)** SRH and ADL scores 2 years after intervention; *: compared with the non-usage group, *p <* 0.05].

The study collected data on the SRH and ADL scores of two groups of chronic disease samples before and after a two-year intervention. Prior to the intervention, the SRH and ADL scores of the usage group were (2.6 ± 1.0) and (65.3 ± 10.3) points, respectively, while those of the non-usage group were (2.4 ± 1.1) and (67.8 ± 9.2) points, respectively. Upon comparison, the SRH and ADL scores differed slightly between the two groups before the intervention (*p* > 0.05) (Figures 2A,B). However, a slight baseline imbalance in ADL scores was observed (SMD = 0.25 > 0.2). To avoid overestimating intervention effects, all subsequent regression models included additional adjustments for baseline ADL, SRH, and CES-D scores. Two years after the intervention, the SRH and ADL scores of the usage group were (4.6 ± 1.2) and (95.9 ± 10.9) points, respectively, while those of the non-usage group were (3.8 ± 1.5) and (84.9 ± 11.0) points, respectively. After baseline adjustment, the intervention group maintained significantly better SRH and ADL outcomes than the non-user group (*p* < 0.05) (Figures 2C,D). Post-intervention ADL scores in the intervention group showed an 11.0-point improvement (95% CI: 9.8–12.2) compared to controls, with a large effect size (Cohen’s d = 1.02). The clinically meaningful improvement (MCID = 10 points) in ADL scores suggests the practical value of smart healthcare for functional recovery.

### 4.4 Changes in psychological health indicators

**Figure 3:**
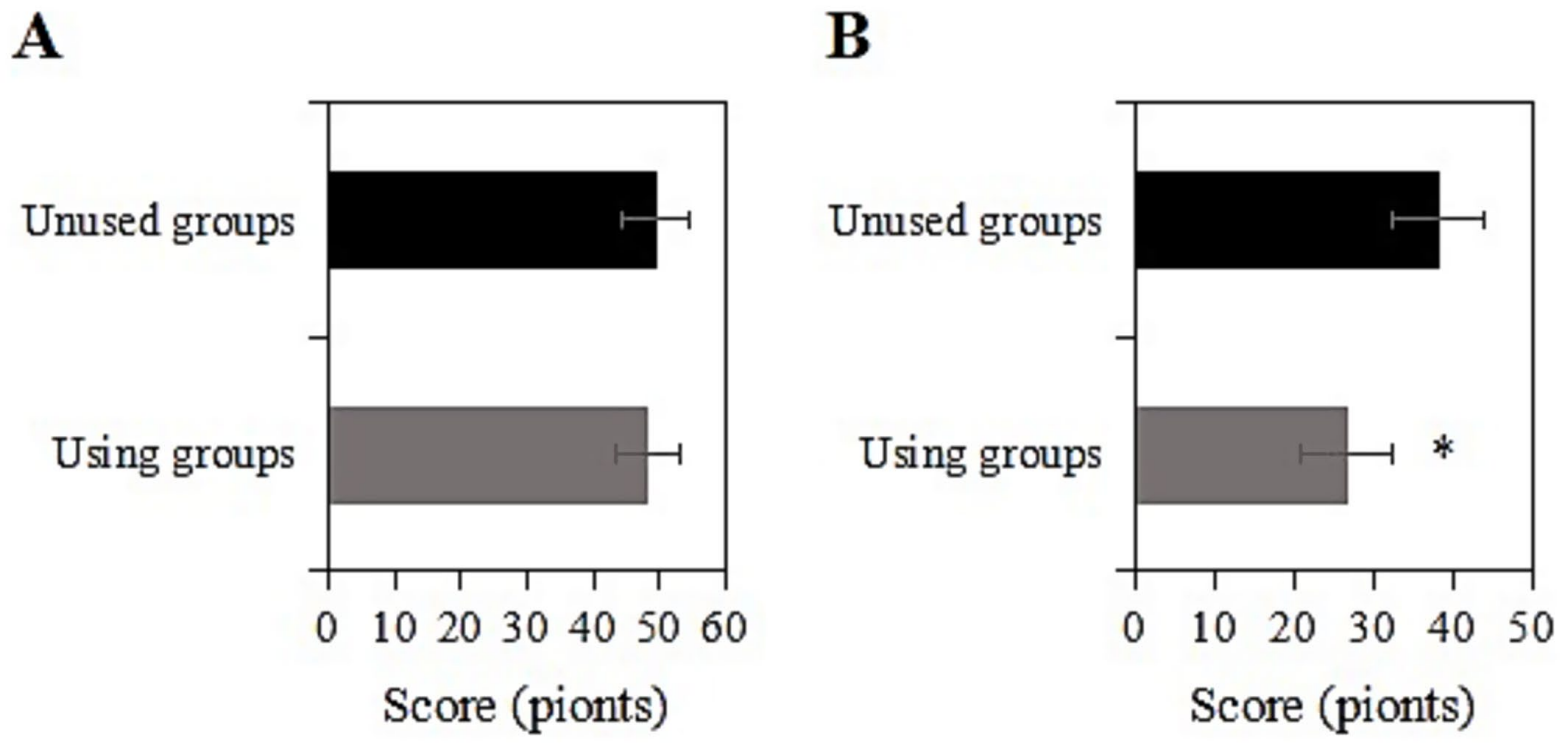
Comparison of CES-D scores before and 2 years after intervention [**(A)** Before intervention; **(B)** 2 years after intervention; *: compared with the non-usage group, *p <* 0.05].

The study collected data on the CES-D scores of two groups of chronic disease samples before and after a two-year intervention. Prior to the intervention, the CES-D score of the usage group was (48.1 ± 10.0) points, while that of the non-usage group was (49.4 ± 9.3) points. Upon comparison, the CES-D scores exhibited inconsiderable differences between the two groups before the intervention (*p* > 0.05) (Figure 3A). Two years after the intervention, the CES-D score of the usage group was (26.6 ± 7.2) points, while that of the non-usage group was (38.2 ± 8.9) points. Upon comparison, after the intervention, the CES-D score of the usage group was notably inferior to that of the non-usage group (*p <* 0.05) (Figure 3B). The usage group demonstrated a significant reduction in CES-D scores by 11.6 points (95% CI: −13.1 to −10.1), with a large effect size (Cohen’s d = 1.31) that exceeded the minimal clinically important difference (MCID = 5 points) for depression scales.

### 4.5 SRH, ADL, and CES-D scores of the usage group under different genders after intervention

**Figure 4:**
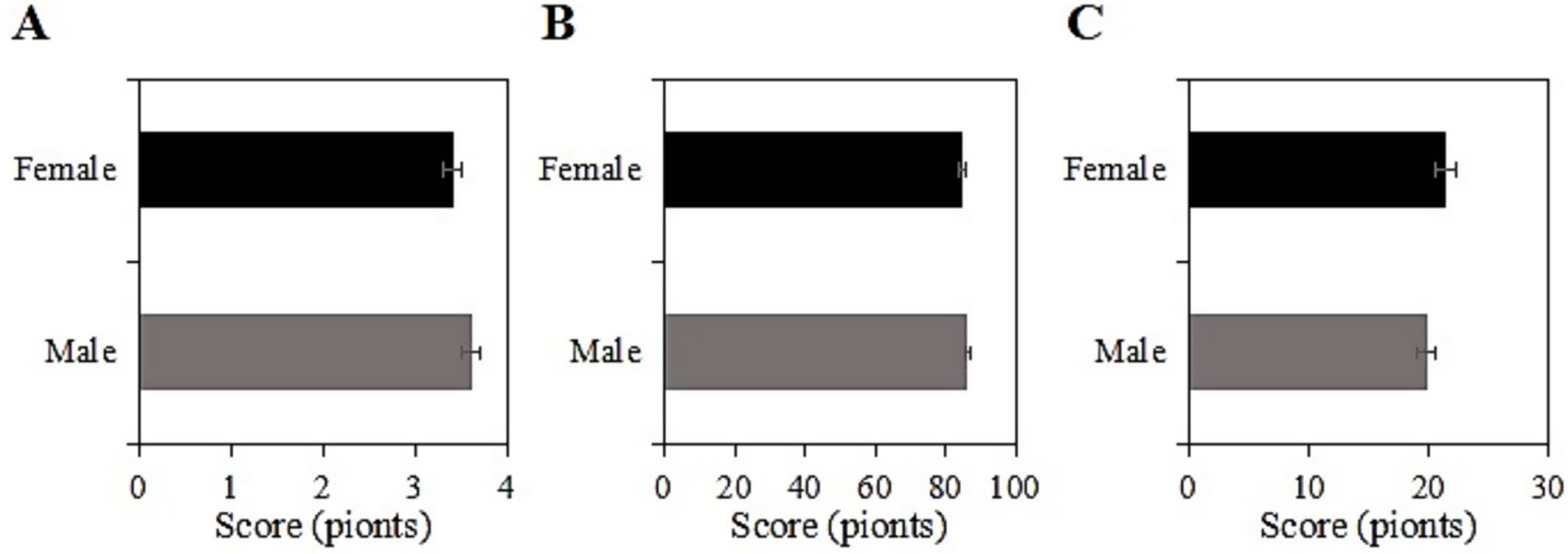
SRH **(A)**, ADL **(B)**, and CES-D **(C)** scores of the usage group under different genders.

The study collected post-intervention data on SRH, ADL, and CES-D scores among male and female participants in the usage group. Among male participants, the SRH, ADL, and CES-D scores were (3.6 ± 1.0), (86.5 ± 10.2), and (19.8 ± 7.2) points, respectively, while among female participants, the scores were (3.4 ± 1.1), (84.8 ± 11.5), and (21.4 ± 8.0) points, respectively. Upon comparison, there were neglectable differences in SRH, ADL, and CES-D scores between male and female participants in this group (*p* > 0.05) (Figure 4).

### 4.6 SRH, ADL, and CES-D scores of the usage group under different age groups after intervention

**Figure 5:**
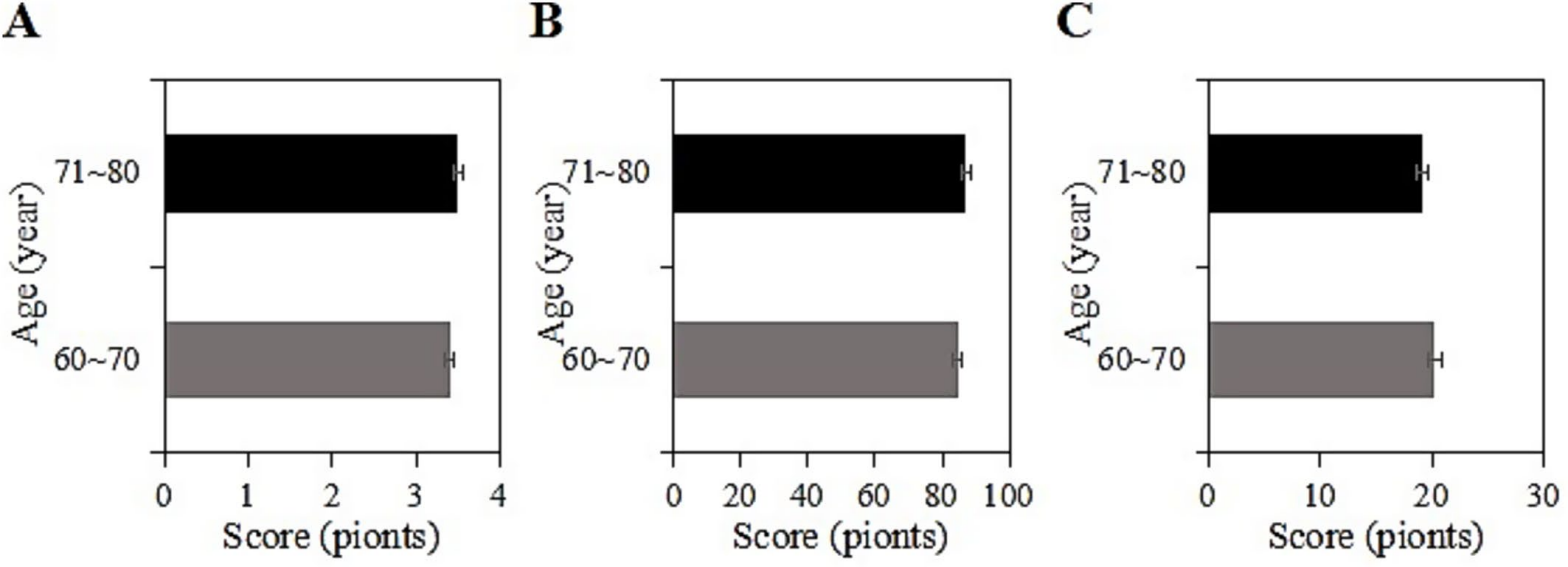
SRH **(A)**, ADL **(B)**, and CES-D **(C)** scores of the usage group under different age groups.

The study collected post-intervention data on SRH, ADL, and CES-D scores among different age groups (60 ~ 70 years, 71 ~ 80 years) in the usage group. Among participants aged 60 ~ 70 years, the SRH, ADL, and CES-D scores were (3.4 ± 1.2), (84.5 ± 10.1), and (20.2 ± 7.4) points, respectively, while among participants aged 71 ~ 80 years, the scores were (3.5 ± 1.0), (87.0 ± 11.1), and (19.1 ± 8.2) points, respectively. Upon comparison, the SRH, ADL, and CES-D scores differed inconsiderably between the two age groups in this group (*p* > 0.05) (Figure 5).

### 4.7 SRH, ADL, and CES-D scores of the usage group under different durations of chronic diseases after intervention

**Figure 6:**
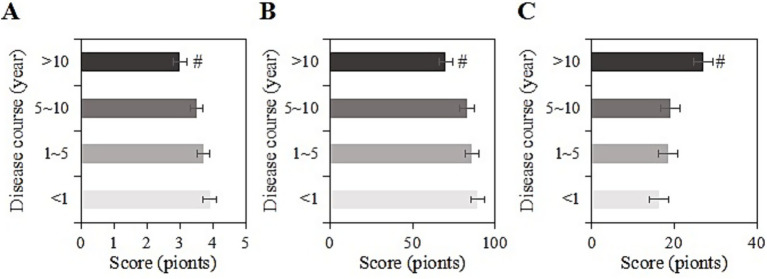
SRH **(A)**, ADL **(B)**, and CES-D **(C)** scores of the usage group under different durations of chronic diseases (#: compared to <1 year, 1–5 years, 5–10 years, *p <* 0.05).

The study collected post-intervention data on SRH, ADL, and CES-D scores among different durations of chronic disease (<1 year, 1 ~ 5 years, 6 ~ 10 years, >10 years) in the usage group. For samples with a duration of <1 year, the SRH, ADL, and CES-D scores were (3.9 ± 1.0), (89.3 ± 11.3), and (16.2 ± 5.0) points, respectively; for those with a duration of 1 ~ 5 years, the scores were (3.7 ± 1.4), (85.9 ± 10.9), and (18.4 ± 8.0) points, respectively; for those with a duration of 5 ~ 10 years, the scores were (3.5 ± 1.0), (83.0 ± 11.2), and (19.1 ± 8.2) points, respectively; and for those with a duration of >10 years, the scores were (3.0 ± 1.0), (70.0 ± 11.1), and (27.0 ± 9.3) points, respectively. Upon comparison, as the duration of the chronic disease increased, SRH and ADL gradually decreased, while CES-D scores increased. Samples with a chronic disease duration of >10 years had the lowest SRH and ADL scores and the highest CES-D score (*p <* 0.05) (Figure 6).

### 4.8 SRH, ADL, and CES-D scores of the usage group under different chronic disease types after intervention

**Figure 7:**
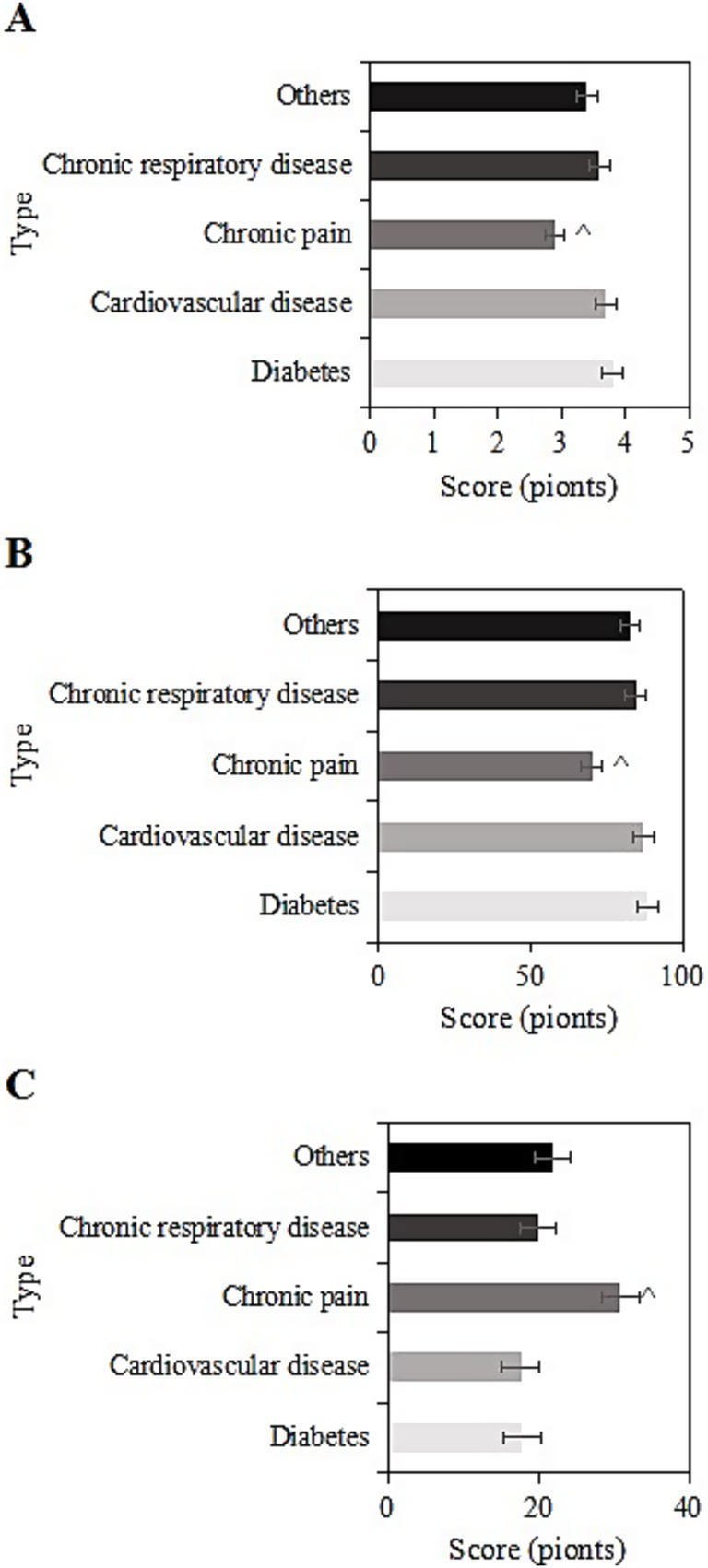
SRH **(A)**, ADL **(B)**, and CES-D **(C)** scores of the usage group under different chronic disease types (^: compared to diabetes, cardiovascular diseases, chronic respiratory system diseases, and other chronic diseases, *p <* 0.05).

The study collected post-intervention data on SRH, ADL, and CES-D scores among different types of chronic diseases (diabetes, cardiovascular diseases, chronic pain, chronic respiratory diseases, other) in the usage group. For samples with diabetes, the SRH, ADL, and CES-D scores were (3.8 ± 1.1), (88.5 ± 10.2), and (17.8 ± 6.3) points, respectively. For cardiovascular diseases, the scores were (3.7 ± 1.8), (87.2 ± 9.2), and (17.6 ± 8.3) points, respectively. For chronic pain, the scores were (2.9 ± 1.0), (70.2 ± 8.2), and (30.9 ± 10.3) points, respectively. For chronic respiratory diseases, the scores were (3.6 ± 1.7), (84.5 ± 11.6), and (20.0 ± 8.6) points, respectively. For other chronic diseases, the scores were (3.4 ± 1.0), (82.8 ± 10.9), and (21.9 ± 8.9) points, respectively. Upon comparison, samples with chronic pain had the lowest SRH and ADL scores and the highest CES-D score (*p <* 0.05) (Figure 7).

### 4.9 SRH, ADL, and CES-D scores of the usage group under different income levels after intervention

**Figure 8:**
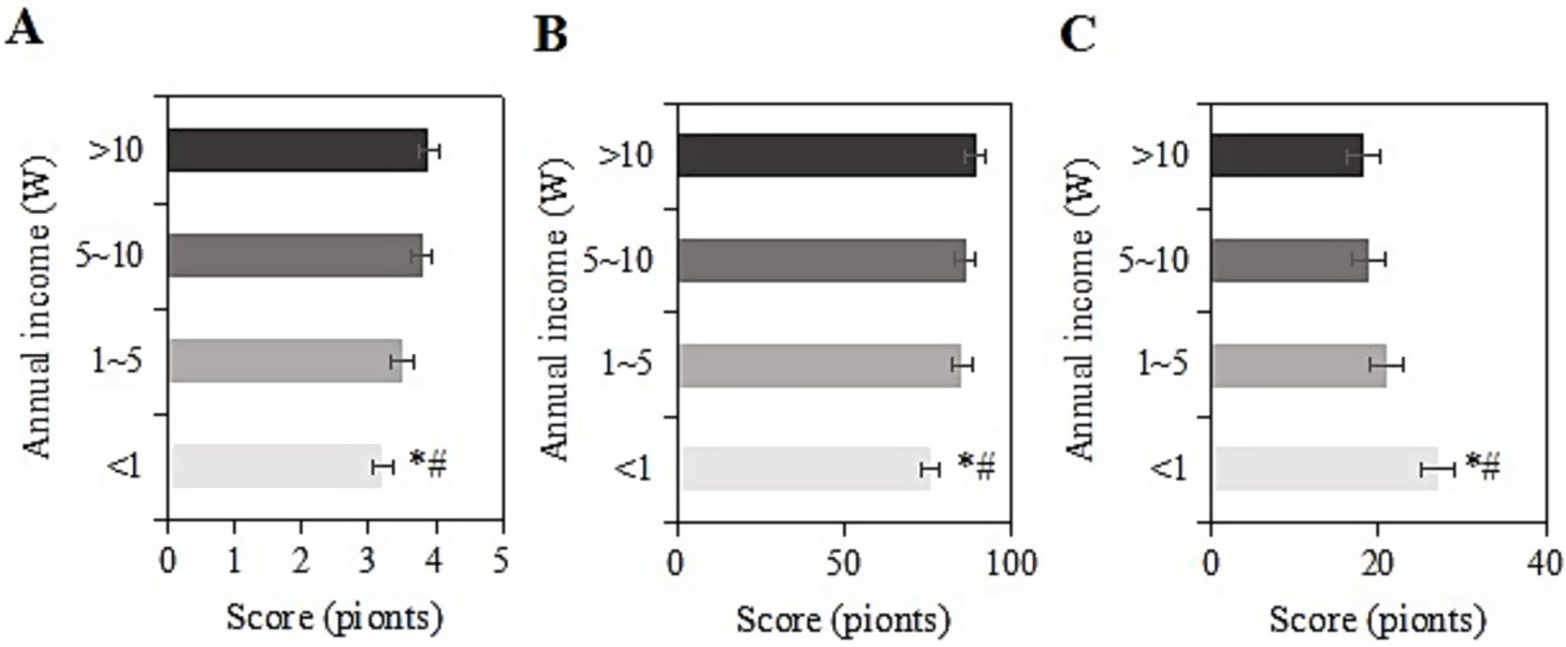
SRH **(A)**, ADL **(B)**, and CES-D **(C)** scores of the usage group under different income levels (*#: compared to 1-5 W, 5-10 W, >10 W, *p <* 0.05).

The study collected post-intervention data on SRH, ADL, and CES-D scores among different income levels (<1 W, 1-5 W, 5-10 W, >10 W) in the usage group. For samples with an income of <1 W, the SRH, ADL, and CES-D scores were (3.2 ± 1.2), (75.9 ± 9.2), and (27.0 ± 6.4) points, respectively. For those with an income of 1-5 W, the scores were (3.5 ± 1.3), (85.2 ± 9.0), and (20.9 ± 8.0) points, respectively. For those with an income of 5-10 W, the scores were (3.8 ± 1.6), (86.1 ± 8.0), and (18.8 ± 8.4) points, respectively. For those with an income of >10 W, the scores were (3.9 ± 1.4), (89.3 ± 10.3), and (18.3 ± 8.4) points, respectively. Upon comparison, as the annual income increased, SRH and ADL scores gradually increased, while CES-D scores gradually decreased. Samples with an income of <1 W had the lowest SRH and ADL scores and the highest CES-D score (*p <* 0.05) (Figure 8).

### 4.10 SRH, ADL, and CES-D scores of the usage group under different education levels after intervention

**Figure 9:**
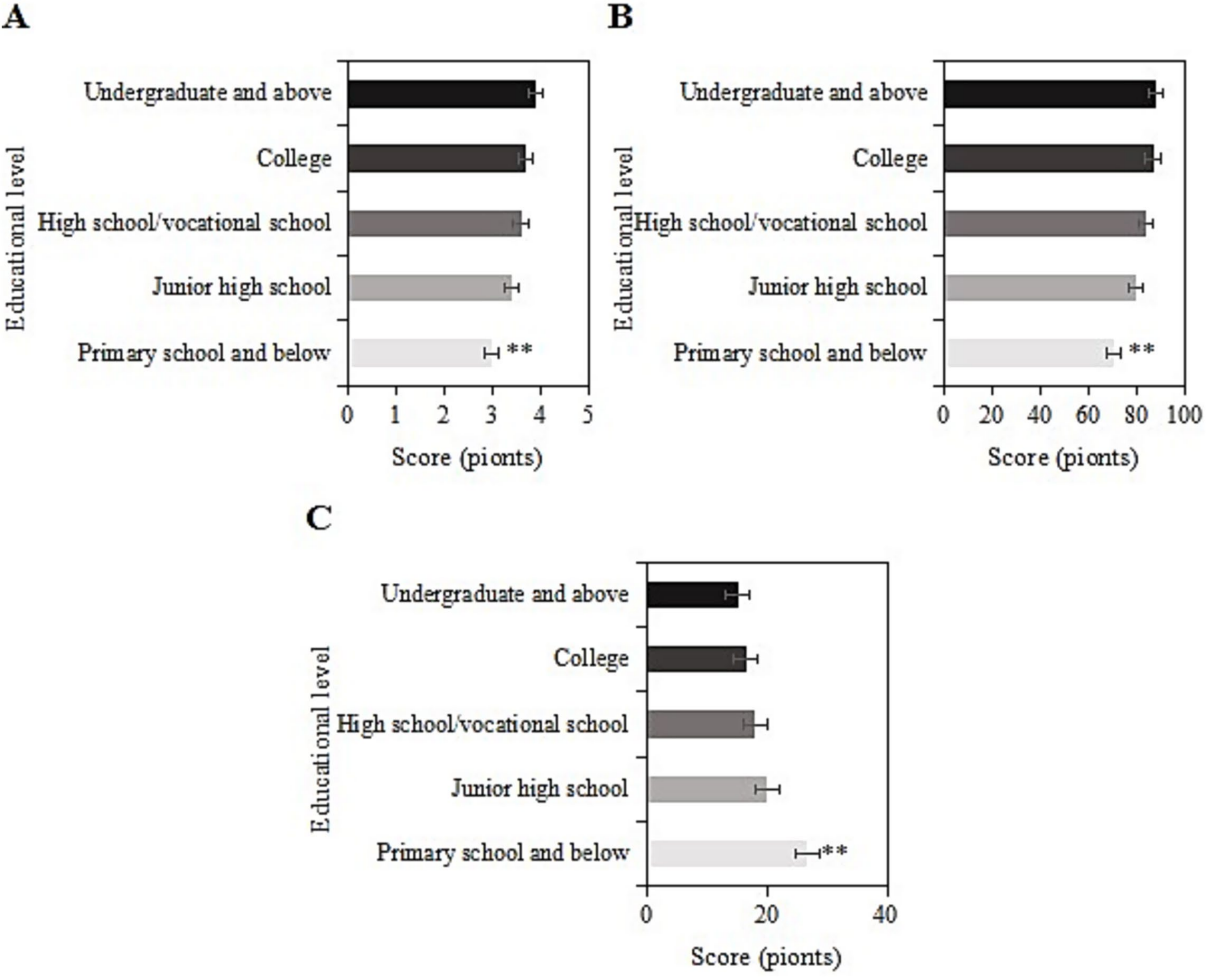
SRH **(A)**, ADL **(B)**, and CES-D **(C)** scores of the usage group under different education levels (**: compared to primary school, junior high school, senior high school/vocational, junior college, and undergraduate and above, *p <* 0.05).

The study collected post-intervention data on SRH, ADL, and CES-D scores among different education levels (elementary school and below, middle school, high school/vocational school, college, and above) in the usage group. For samples with an education level of elementary school and below, the SRH, ADL, and CES-D scores were (3.0 ± 1.1), (70.7 ± 9.1), and (26.7 ± 6.0) points, respectively. For those with a middle school education, the scores were (3.4 ± 1.9), (80.0 ± 9.7), and (20.1 ± 8.3) points, respectively. For those with a high school/vocational school education, the scores were (3.6 ± 1.3), (84.1 ± 5.8), and (18.0 ± 8.6) points, respectively. For those with a college education, the scores were (3.7 ± 1.0), (86.9 ± 9.4), and (16.6 ± 7.3) points, respectively. For those with a bachelor’s degree or above, the scores were (3.9 ± 1.1), (88.0 ± 11.4), and (15.2 ± 7.1) points, respectively. Upon comparison, as the education level increased, SRH and ADL scores gradually increased, while CES-D scores gradually decreased. Samples with an education level of elementary school and below had the lowest SRH and ADL scores and the highest CES-D score (*p <* 0.05) (Figure 9).

### 4.11 SRH, ADL, and CES-D scores of the usage group under different smart healthcare service types after intervention

**Figure 10:**
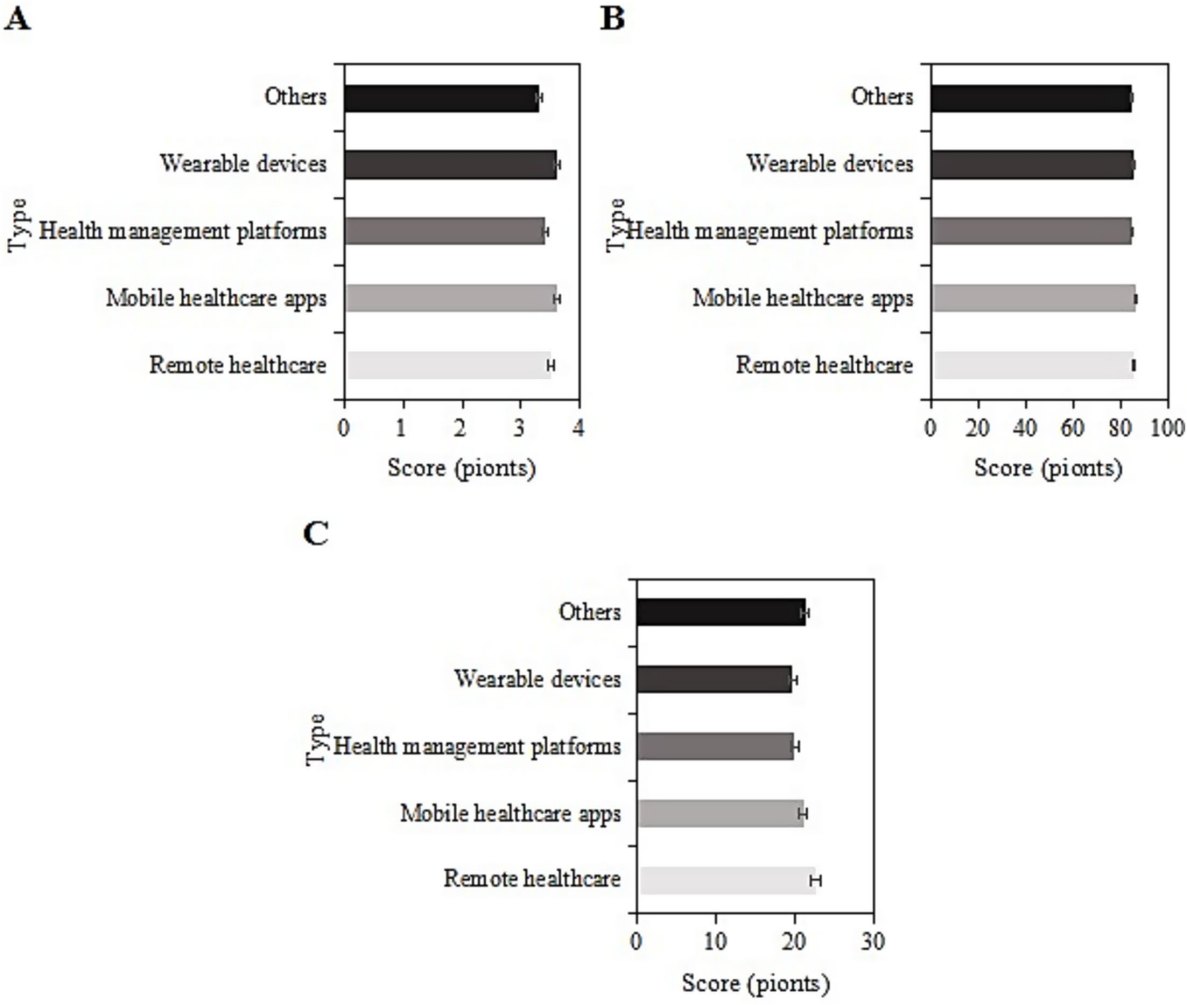
SRH **(A)**, ADL **(B)**, and CES-D **(C)** scores of the usage group under different smart healthcare service types.

The study collected post-intervention data on SRH, ADL, and CES-D scores among different types of smart healthcare services (remote medical services, mobile medical apps, health management platforms, wearable devices, and others) in the usage group. For samples using remote medical services, the SRH, ADL, and CES-D scores were (3.5 ± 1.0), (85.4 ± 9.2), and (22.5 ± 6.2) points, respectively. For those using mobile medical apps, the scores were (3.6 ± 1.5), (86.9 ± 9.1), and (20.9 ± 8.4) points, respectively. For those using health management platforms, the scores were (3.4 ± 1.0), (84.6 ± 9.4), and (19.8 ± 8.3) points, respectively. For those using wearable devices, the scores were (3.6 ± 1.1), (86.1 ± 9.0), and (19.6 ± 7.8) points, respectively. For the “other” category, the scores were (3.3 ± 1.2), (84.8 ± 10.2), and (21.2 ± 8.3) points, respectively. Upon comparison, the SRH, ADL, and CES-D scores differed slightly among the different types of smart healthcare services in this group (*p* > 0.05) (Figure 10).

### 4.12 Logistic regression analysis

Based on the above results, logistic regression analysis was further conducted to examine the effects of disease duration, type of chronic disease, income level, and educational level on the SRH, ADL, and CES-D scores of chronic disease patients assisted by Internet-based smart healthcare services. The results are presented in Table 2. A disease duration of over 10 years and chronic pain were risk factors affecting SRH, ADL, and CES-D scores. Additionally, an income level of less than 1 W and an educational level of primary school or below were risk factors for SRH scores (*p <* 0.05).

**Table 2:** Logistic regression analysis.

Dependent variable	*t*	*p*	OR	95%*CI*
SRH				
Chronic disease course (>10 years)	−2.10	0.04*	3.45	1.12–5.78
Chronic pain	1.98	0.05*	3.18	1.12–4.98
Income level (<10,000)	2.62	0.03*	0.85	0.51–1.38
Primary school education and below	2.32	0.02*	1.15	0.98–1.35
ADL				
Chronic disease course (>10 years)	−2.05	0.01*	3.21	1.18–5.98
Chronic pain	1.98	0.03*	3.18	1.12–4.98
CES-D				
Chronic disease course (>10 years)	2.08	0.02*	3.32	1.08–5.66
Chronic pain	1.95	0.03*	3.12	1.02–4.95

*p < 0.05.

### 4.13 Endogeneity treatment and robustness check results

To validate the robustness of the regression estimates and the plausibility of causal inference, this study further incorporates instrumental variable methods (2SLS), panel fixed-effects models, and various robustness checks.

The results of the instrumental variable analysis indicate that the instrumental variables are significantly correlated with the use of smart healthcare (First-stage *F* value = 32.6, *p* < 0.01), and the Hansen J test shows *p* = 0.21, supporting the exogeneity of the instruments. The 2SLS results reveal that the effects of smart healthcare use on SRH (*β* = 0.38, *p* = 0.02), ADL (*β* = 4.12, *p* = 0.01), and CES-D (*β* = −2.95, *p* = 0.03) are consistent with the direction found in the logistic regression, and the effect sizes are larger.

The panel fixed-effects model, after controlling for individual fixed effects, shows that the use of smart healthcare still significantly improves SRH (*β* = 0.25, *p* = 0.04) and ADL (*β* = 3.78, *p* = 0.02), and reduces CES-D (*β* = −2.01, *p* = 0.03).

The robustness check results indicate that after replacing variables, smart healthcare has a significant negative impact on the incidence of complications (OR = 0.76, *p* = 0.04). Subgroup regressions show consistent results across urban–rural and gender subgroups. After PSM matching, significant differences in SRH, ADL, and CES-D between the treatment and control groups remain (*p* < 0.05). Detailed results are presented in Table 3.

**Table 3:** Summary of instrumental variable and robustness check results.

Analysis method	SRH (β/OR)	ADL (β/OR)	CES-D (β/OR)	*p*
2SLS	0.38	4.12	−2.95	0.02
Panel fixed effects	0.25	3.78	−2.01	0.03
PSM matching	1.45*	1.62*	0.68*	<0.05

PSM results are presented as OR values; β represents standardized coefficients.

## 5 Discussion

Chronic diseases represent one of the most significant health challenges today. These conditions are characterized by their long-term persistence, often being incurable, and typically involve a sustained or gradual progression that may last for months or even years. Chronic diseases include, but are not limited to, hypertension, diabetes, heart disease, chronic obstructive pulmonary disease, chronic pain, and others (11, 12). These conditions typically have profound effects on the quality of life and overall health status of patients. Aguilar et al. (13) proposed that asthma affects the physical and mental health as well as the social and emotional development of patients. Rezaei-Tavabe et al. (14) suggested that older adults individuals with chronic diseases are more susceptible to other complications. Sarbazi et al. (15) suggested that compared to healthy individuals, individuals with type 2 diabetes experience a significant decline in physical functioning, a decrease in quality of life, and an increase in negative psychological symptoms. Xia et al. (16) pointed out that risk factors for anxiety symptoms are associated with chronic diseases. It is evident that chronic diseases not only have negative effects on the physical health of patients but also adversely affect their mental health. In recent years, with the deepening implementation of the “Internet Plus” strategy, smart healthcare, as a product combining Internet technology and medical health services, has gradually become an important approach to address the management of chronic diseases in the older adults.

Currently, the application of Internet Plus smart healthcare technology in the management of chronic diseases has made significant progress. A substantial body of research has demonstrated that Internet Plus smart healthcare technology holds promising potential in the management of chronic diseases through various aspects, including remote monitoring, health management platforms, AI and big data analysis, remote diagnosis and treatment, and patient education. For instance, Guo and Lyu (17) also applied mobile healthcare to chronic cardiovascular diseases, such as the management of hypertension. Dhamanti et al. (18) confirmed that smart healthcare can promote health by monitoring health conditions, recording physical activities and rehabilitation progress, and improving overall quality of life. The study collected data from older adults chronic disease samples in the 2023 CFPS dataset for analysis. It was found that after intervention, the SRH and ADL of the usage group samples were higher than those of the non-usage group, and the CES-D scores were markedly inferior to those of the non-usage group (*p <* 0.05). This further confirms that with the assistance of “Internet Plus” smart healthcare technology, the physical and mental health of patients with chronic diseases are more likely to improve.

It has been suggested that factors such as gender, income level, and educational level may influence the effectiveness of Internet Plus smart healthcare technology in improving the physical and mental health of patients with chronic diseases (19). Building upon this, logistic regression analysis was conducted in this study, and the results revealed that chronic disease duration >10 years and chronic pain would decrease patients’ ADL and increase CES-D scores. Moreover, chronic disease duration >10 years, chronic pain, income level <1 W, and education level of primary school and below would all decrease SRH scores. Longer and more recurrent chronic diseases exacerbate patients’ suffering, both physically and economically, leading to an increased psychological burden. Chronic pain refers to continuous or intermittent pain lasting more than 3 months. Long-term chronic pain not only leads to a decline in patients’ physical function but may also result in drug dependence or addiction. Pain can also affect patients’ sleep, leading to a decrease in their quality of life and triggering emotional disorders such as depression and anxiety (20). Therefore, a longer duration of chronic illness and persistent chronic pain significantly impair patients’ physical and mental health. Patients with an annual income level <1 W belong to the low-income group, and the development of smart medical platforms involves relatively high costs (21), leading to the existence of fee-based services in most cases. For instance, in some online diagnosis and treatment platforms of internet hospitals, text-based consultations cost 0–50 yuan per session, while video consultations cost 50–100 yuan per session. Chronic illness, being a long-term and incurable condition, constitutes sustained expenses. Additionally, chronic patients require long-term medication. Therefore, for low-income individuals, smart medical services are not readily available, making it difficult to seek timely consultation for disease management, which is not conducive to improving their condition. Moreover, most chronic patients over the age of 60 with only primary school education have limited literacy and face difficulties in using Internet-based intelligent medical services. They often need assistance from their children, resulting in inadequate disease management. Considering the aforementioned issues, it is essential to reduce costs as much as possible in the development of smart medical services, making it easier to minimize charges for users in the future. Furthermore, it is crucial to promote the use of smart medical platform operations among low-educated groups at lower price points.

This observational study employed natural grouping (non-random allocation) based on “smart healthcare technology usage” among older adults chronic disease patients, potentially introducing self-selection bias (e.g., individuals with higher education or income may be more likely to voluntarily adopt smart healthcare technologies). To minimize such bias, we incorporated instrumental variable (IV) analysis, propensity score matching (PSM), and panel data models for robustness verification. However, the exogeneity of instrumental variables may remain limited (e.g., whether community smart healthcare demonstration sites can fully account for unobserved confounders). Future studies could employ natural experiments or randomized controlled trials (RCTs) to further validate the causal effects of smart healthcare on patients’ physical and mental health, enabling more rigorous assessment of long-term impacts. Compared with existing research, this study innovatively addresses endogeneity issues, with findings demonstrating robust correlations across different models. These results are consistent with the findings of Shagerdi et al. (22) and Jiang et al. (23), indicating that smart healthcare has a sustained impact on improving patients’ physical and mental health by enhancing health management capabilities and healthcare accessibility. Notably, the instrumental variable analysis reveals that regional internet penetration and the presence of community smart healthcare demonstration sites significantly promote the use of smart healthcare among older adults patients, suggesting that policymakers should strengthen digital infrastructure at the grassroots level, particularly in low-income and low-education regions. Furthermore, results from panel data models demonstrated progressively strengthened associations between smart healthcare utilization and health improvements over time, supporting its research value as a potentially sustainable solution for chronic disease management. However, this study has certain limitations, such as the potential inability of the selected instrumental variables to fully eliminate the influence of unobservable confounding factors. Although PSM matching balances observable variables, it cannot address latent biases. Future research could employ natural experiments or randomized controlled trials to further validate the causal effects.

## 6 Conclusion

Analysis of CFPS data revealed significant associations between “Internet+”-based smart healthcare utilization and improved physical/mental health outcomes among older adults chronic disease patients. However, it is crucial to consider the impact of factors such as the prolonged duration of chronic illness, chronic pain, low income, and low education levels on the effectiveness of smart healthcare interventions. Subsequent research should further explore and analyze improvements in these areas. Additionally, although the study has achieved a reasonable breadth in sample collection, the basic data analysis is not sufficiently comprehensive. Factors such as patients’ living conditions were not considered, and an analysis of the impact of these basic conditions on the frequency of Internet Plus smart medical application was not conducted. Further research should strengthen the analysis of this aspect to make the study more comprehensive.

## Data Availability

The data analyzed in this study is subject to the following licenses/restrictions: The data are from China Family Panel Studies (CFPS), funded by Peking University and the National Natural Science Foundation of China, and maintained by the Institute of Social Science Survey of Peking University. Restrictions apply to the availability of these data, which were used under license for this study. Requests to access these datasets should be directed to CFPS, Peking University, https://opendata.pku.edu.cn/.
